# O6-8 Rethinking schools as a setting for physical activity promotion in the 21st Century - A position paper from the Erasmus+ 2PASS 4Health project

**DOI:** 10.1093/eurpub/ckac094.048

**Published:** 2022-08-29

**Authors:** Enrique Garcíá Bengoechea, Elaine Murtagh, Caera Grady, Julien Bois, Nicolas Fabre, Alberto Aibar Solano, Lionel Dubertrand, Maïté Verloigne, Jose Ribeiro, Catherine B Woods

**Affiliations:** 1 Visiting fellow, Physical Education and Sports Sciences Department, University of Limerick, Limerick, Ireland; 2 Coordinator of the Irish Physical Activity Research Collaboration, Sport Ireland, Dublin, Ireland; 3 Physical Education and Sports Science, University of Limerick, Limerick, Ireland; 4 Department of Science and Techniques of physical activity and sports activities, University de PAU et Pays de l'Adour, Tarbes, France; 5 Department of Musical, plastic and corporal expression, University of Zaragoza, Zaragoza, Spain; 6 CAPAS-Cité, City of Tarbes, Tarbes, France; 7 Department of public health and primary care, University of Gent, Ghent, Belgium; 8 Faculty of Sport, University of Porto, Porto, Portugal

**Keywords:** Adolescents, Whole-of-school programmes, systems approach

## Abstract

**Background:**

Schools are ideally placed to provide children and adolescents with multiple opportunities to be or learn to be physically active. However, key reviews have reported that interventions to date have largely failed to have any long-term impact on overall physical activity levels. In this position paper, greater attention to key issues is needed to realise the full potential of schools and ideal physical activity for health promotion setting.

**Methods:**

This study draws on multi-author expertise to develop a position paper to advance opinion on school-based programmes. Collaborative conceptual thinking was established through various tools such as literature review, evidence synthesis and online and in person meetings.

**Results/Discussion:**

The adoption of a systems approach is valuable for understanding the complexities of the school setting and to support the implementation of whole-of-school initiatives. Furthermore, we contend that the full range of physical, cognitive, emotional and social benefits that physical activity provides should be considered, rather than a narrow focus solely on physical activity levels. Interdisciplinary research questions are most useful in exploring and evaluating whole-of-school approaches. Informed by process, impact and outcome evaluation and implementation science, both qualitative and quantitative research methodologies and a move beyond traditional research design are needed to advance our knowledge of what works, for whom and in what context. Case studies from several European countries will be presented to illustrate examples of systems approaches in action. This includes examples at multiple levels firstly, a national approach including a Physical Education curriculum reformation (Portugal), a regional approach such as a county council partnership with a University to support physical activity promotion (France) and a local approach at the school level i.e. a whole-of-school physical activity programme (Spain and Ireland).

**Conclusion:**

From authors expertise and reflection, this paper makes recommendations on the nature of the evidence required to bridge the implementation gap, sustain and scale-up innovative approaches to whole-of-school programmes.

